# Rheumatoid Arthritis Synovial Fluid Neutrophils Drive Inflammation Through Production of Chemokines, Reactive Oxygen Species, and Neutrophil Extracellular Traps

**DOI:** 10.3389/fimmu.2020.584116

**Published:** 2021-01-05

**Authors:** Helen L. Wright, Max Lyon, Elinor A. Chapman, Robert J. Moots, Steven W. Edwards

**Affiliations:** ^1^ Institute of Life Course and Medical Sciences, University of Liverpool, Liverpool, United Kingdom; ^2^ Department of Rheumatology, Aintree University Hospital, Liverpool, United Kingdom; ^3^ Faculty of Health, Social Care and Medicine, Edge Hill University, Ormskirk, United Kingdom; ^4^ Institute of Infection, Veterinary and Ecological Sciences, University of Liverpool, Liverpool, United Kingdom

**Keywords:** neutrophils, rheumatoid arthritis, synovial fluid, transcriptomics, neutrophil extracellular traps

## Abstract

Rheumatoid arthritis (RA) is a chronic inflammatory disorder affecting synovial joints. Neutrophils are believed to play an important role in both the initiation and progression of RA, and large numbers of activated neutrophils are found within both synovial fluid (SF) and synovial tissue from RA joints. In this study we analyzed paired blood and SF neutrophils from patients with severe, active RA (DAS28>5.1, n=3) using RNA-seq. 772 genes were significantly different between blood and SF neutrophils. IPA analysis predicted that SF neutrophils had increased expression of chemokines and ROS production, delayed apoptosis, and activation of signaling cascades regulating the production of NETs. This activated phenotype was confirmed experimentally by incubating healthy control neutrophils in cell-free RA SF, which was able to delay apoptosis and induce ROS production in both unprimed and TNFα primed neutrophils (p<0.05). RA SF significantly increased neutrophil migration through 3μM transwell chambers (p<0.05) and also increased production of NETs by healthy control neutrophils (p<0.001), including exposure of myeloperoxidase (MPO) and citrullinated histone-H3-positive DNA NETs. IPA analysis predicted NET production was mediated by signaling networks including AKT, RAF1, SRC, and NF-κB. Our results expand the understanding of the molecular changes that take place in the neutrophil transcriptome during migration into inflamed joints in RA, and the altered phenotype in RA SF neutrophils. Specifically, RA SF neutrophils lose their migratory properties, residing within the joint to generate signals that promote joint damage, as well as inflammation *via* recruitment and activation of both innate and adaptive immune cells. We propose that this activated SF neutrophil phenotype contributes to the chronic inflammation and progressive damage to cartilage and bone observed in patients with RA.

## Introduction

Rheumatoid arthritis (RA) is an inflammatory disorder characterized by systemic inflammation, including swelling and pain in synovial joints. Left untreated, uncontrolled inflammation will destroy joints, causing deformity and disability. Many studies have shown that blood neutrophils have an aberrant, activated phenotype in RA, characterized by increased production of ROS and cytokines, and delayed apoptosis (1–4). As well as having an activated phenotype in peripheral blood, activated neutrophils are found at high numbers in both synovial joints and tissues of patients with RA (5–7). Their presence within RA joints is accompanied by high levels of neutrophil granule proteins in synovial fluid, including myeloperoxidase (MPO), cathepsin G, proteinase 3, elastase, and lactoferrin (1, 8–12). These granule proteins contribute to the pathogenesis of RA through proteolytic cleavage and activation of proteins (including cytokines and chemokines), cleavage of soluble receptors to initiate trans-signaling (such as the IL-6 receptor) and degradation of cartilage (e.g. cleavage of collagen fibers) (1, 13–16). *Ex vivo* synovial fluid (SF) neutrophils have an altered phenotype compared to paired blood neutrophils. They produce higher levels of superoxide $\left(O_{2}^{\cdot -}\right)$ and contain phosphorylated p47^phox^, indicating assembly and activation of the NADPH oxidase (NOX2) *in vivo* (17). They also express the high-affinity FcγR1 receptor (CD64) and MHC Class II proteins (18–21). SF neutrophil lysates also have lower levels of granule proteins such as MPO, indicating they have undergone degranulation within the synovial joint (8). Animal studies and human case studies of early RA suggest an important role for neutrophils in the initiation of synovial inflammation in RA joints (5, 7, 22), possibly through the release of granule enzymes and production of VEGF, both of which enable fibroblast adhesion and growth of the inflammatory synovial pannus (23, 24). A key role for exposure of citrullinated antigens on neutrophil extracellular traps (NETs) has been proposed in the initiation of auto-immunity and development of anti-citrullinated peptide auto-antibodies (ACPA) in RA (1, 25). NET products are detectable in both RA serum and synovial fluid (12, 26) and NETs have been observed in RA synovial biopsy tissues by staining for CD15, elastase, MPO and citrullinated (cit) histone H3 (25, 27). It was recently shown that up to 70% of newly-diagnosed RA patients have auto-antibodies in their serum that recognize NET components (ANETA) (28).

We have extensively studied neutrophil phenotype in RA and shown that RA neutrophils have activated NF-κB signaling leading to delayed apoptosis (2). Additionally, we have shown that the neutrophil transcriptome is altered in RA compared to healthy controls (29) and that gene expression in RA patients pre-TNFi therapy can be used to stratify patients based on response or non-response to therapy (30). Whilst several studies have analyzed SF neutrophil functions, to our knowledge none have measured the transcriptome of RA SF neutrophils compared to paired blood neutrophils. In this study we first used RNA-seq to describe the changes that take place when blood neutrophils migrate into RA joints and then validated our bioinformatics predictions using healthy control neutrophils incubated in RA SF *in vitro*. We show that RA SF neutrophils have an altered phenotype, including decreased expression of genes associated with extravasation and migration, increased expression of chemokines, FcγR1 and MHC II, decreased apoptosis and increased ROS and NET production. We propose that these altered properties enable SF neutrophils to regulate inflammatory events by attracting and activating innate and adaptive immune cells, including other neutrophils, T cells, NK cells, monocytes, macrophages, and dendritic cells within diseased RA joints (31–33).

## Methods

### Ethics Statement and Patient Selection

This study was approved by the University of Liverpool Committee on Research Ethics for healthy controls, and NRES Committee North West (Greater Manchester West, UK) for RA patients. All participants gave written, informed consent in accordance with the declaration of Helsinki. All patients fulfilled the ACR 2010 criteria for the diagnosis of RA (34).

### Blood and Synovial Fluid Collection

Peripheral blood was drawn into heparinized vacutainers from healthy controls and RA patients. Synovial fluid was aspirated from the knee joint of RA patients (n=3) approximately 1 month prior to the start of the TNFi therapy Etanercept. All patients had a DAS28 score greater than 5.1 at the time of sample collection. Patients had a mean age of 59 years and an average disease duration of 20.6 years; two patients were female (**Supplementary Table 1**). Synovial fluid was decanted into universal tubes containing 50 μl heparin immediately upon collection and neutrophils were isolated within 1 h. Aliquots of un-diluted synovial fluid were centrifuged at 2000 g for 5 min to remove leukocytes. Cell-free synovial fluid was decanted and frozen at -20°C.

### Neutrophil Isolation

Neutrophils were isolated from heparinized peripheral blood using Polymorphprep (Axis Shield) as previously described (2, 35). Erythrocytes were lysed using hypotonic lysis with ammonium chloride buffer. Synovial fluid was passed through gauze prior to dilution 1:1 with PBS. Neutrophils were isolated from diluted synovial fluid using Ficoll-Paque (GE Healthcare). Neutrophil purity from both peripheral blood and synovial fluid isolations was >97% as assessed by cytospin (**Supplementary Figure 1**). Neutrophils accounted for an average of 85.7% leukocytes in whole synovial fluid as assessed by cytospin (**Supplementary Figure 1**, **Supplementary Table 1**). Following isolation, neutrophils were resuspended in RPMI 1640 media (Life Technologies) containing L-glutamine (2 mM) and Hepes (25 mM) at a concentration of 5x10^6^/ml.

### RNA Extraction

RNA was isolated from 10^7^ neutrophils using Trizol-chloroform (Life Technologies), precipitated in isopropanol and cleaned using the RNeasy kit (Qiagen) including a DNase digestion step. RNA was snap-frozen in liquid nitrogen and stored at -80°C. Total RNA concentration and integrity were assessed using the Agilent 2100 Bioanalyzer RNA Nano chip. RNA integrity (RIN) was ≥ 7.0.

### RNA Sequencing

Total RNA was enriched for mRNA using poly-A selection. Standard Illumina protocols were used to generate 50 base pair single-end read libraries. Libraries were sequenced on the Illumina HiSeq 2000 platform. Reads were mapped to the human genome (hg38) using TopHat v2.0.4 (36) and gene expression (RPKM) values were calculated using Cufflinks v2.0.2 (37). A minimum RPKM threshold of expression of ≥ 0.3 was applied to the data in order to minimize the risk of including false positives against discarding true positives from the dataset (35, 38). Statistical analysis of differential gene expression was carried out using Cuffdiff (a program within Cufflinks) applying a 5% false discovery rate (FDR) for significance. The raw sequencing data reported in this manuscript have been deposited in the NCBI Gene Expression Omnibus (GEO) and are accessible through GEO Series accession number GSE154474.

### Bioinformatics Analysis

Bioinformatics analysis was carried out using IPA (Ingenuity^®^ Systems, www.ingenuity.com), as previously described, applying a 1.5-fold change in gene expression cut-off and a Benjamini-Hochberg correction to p-values for canonical pathway and upstream regulator analysis. Heatmaps were produced using MeV (39) with Euclidean clustering and average linkage. Multivariate partial least squares-discriminant analysis (PLS-DA) was performed on count data, generated using Rsubread (40) using mixOmics v6.10.9 (41) working in R v 3.6.3. Modular analysis of gene expression was carried out using the CRAN package *tmod* (42) and based on the modular framework for classifying blood genomics studies proposed by Chaussabel and colleagues (43).

### Measurement of Apoptosis

Neutrophils (5x10^5^/ml) were incubated at 37°C in 5% CO_2_ in RPMI 1640 (+Hepes, +L-glutamine) for 18 h in the absence or presence of 10% AB serum (Sigma) or 10% cell-free synovial fluid. Following incubation, 2.5x10^4^ cells were diluted in 50 μl of HBSS (Life Technologies) containing 0.5 μl Annexin V-FITC (Life Technologies), and incubated in the dark at room temperature for 15 min. The total volume was then made up to 500 μl with HBSS containing propidium-iodide (PI, 1 μg/ml, Sigma Aldrich) before analysis by flow cytometry (>5,000 events analyzed) using a Guava EasyCyte flow cytometer.

### Measurement of ROS Production

Neutrophils (5x10^6^/ml) were incubated with or without TNFα (10 ng/ml, Merck) for 20 min. Following priming, neutrophils (2.5x10^5^) were diluted in HBSS in the presence of 10µM luminol (Sigma Aldrich). ROS production was stimulated by either f-Met-Leu-Phe (fMLP, 10^-6^M, Sigma Aldrich) or 25% cell-free synovial fluid. Luminol-enhanced chemiluminescence was measured continuously for 60 min on a Tecan plate reader.

### Chemotaxis Assay

The chemotaxis assay was carried out in 24-well tissue culture plates (coated with 12 mg/ml poly-hema to prevent cell adhesion) using hanging chamber inserts with a 3 μM porous membrane (Merck), as previously described (44). Media containing fMLP (10^-8^M), interleukin-8 (100 ng/ml, Sigma) or 25% (v/v) synovial fluid was added to the bottom chamber. Neutrophils (10^6^/ml) were added to the top chamber and incubated for 90 min at 37°C and 5% CO_2_. The number of migrated cells after 90 min incubation was measured using a Coulter Counter Multisizer-3 (Beckman Coulter).

### Assay for Neutrophil Extracellular Trap Production

Neutrophils were seeded (at 2x10^5^ cells/500 μl) in RPMI media plus 2% AB serum in a 24-well plate containing poly-L-lysine coated coverslips, as previously described (45). Cells were allowed to adhere for 1 h prior to stimulation with phorbol 12-myristate 13-acetate (PMA, 50 nM, Sigma), A23187 (3.8 μM, Sigma) or 10% RA SF. Cells were incubated for a further 4 h to allow for NET production. Cells adhered to coverslips were fixed with 4% paraformaldehyde prior to immunofluorescent staining. Briefly, coverslips were removed from the plate and washed with PBS, permeabilized with 0.05% Tween 20 in TBS, fixed with TBS (2% BSA) and then stained for 30 min on drops of TBS (2% BSA) on parafilm stretched across a clean 24-well plate. Primary antibodies used were mouse anti-myeloperoxidase (1:1000, Abcam) and rabbit anti-citrullinated histone H3 (1:250, Abcam). Coverslips were washed 3 times with TBS prior to secondary antibody staining (anti-rabbit AlexaFluor488, 1:2000, anti-mouse AlexaFluor647, Life Technologies) in TBS (+2% BSA) for 30 min. Coverslips were washed prior to staining with DAPI (1 μg/ml, Sigma). Slides were imaged on an Epifluorescence microscope (Zeiss) using a 40X objective. Images were analyzed using ImageJ (46) and are presented with equal color balance.

### Measurement of Cytokines in Synovial Fluid

The concentrations of granulocyte-colony stimulating factor (GCSF), interferon-γ (IFNG), interleukin-1β (IL1B), interleukin-6 (IL6), and tumor necrosis factor-α (TNF) in RA SF were measured as part of a previous study (47). Briefly, cell-free SF was pre-incubated with blocking buffer (40% mouse serum, 20% goat serum, 20% rabbit serum, all Sigma) for 30 min at room temperature and then centrifuged at 14,000g for 10 min to remove immune complexes. The multiplex cytokine assay (Biosource) was carried out in a 96-well filter plate as per manufacturer’s instructions and as previously described (47). The plate was read in a Bio-Plex Suspension Array system, model Luminex 100 (Bio-Rad), and cytokine concentrations calculated by reference to a standard curve.

### Statistical Analysis

Statistical analysis of experimental data was performed using a Student’s t-test or ANOVA in GraphPad Prism (v5) as stated in the text.

## Results

### Transcriptomic Alterations From Blood to Synovial Fluid

**Figure 1:**
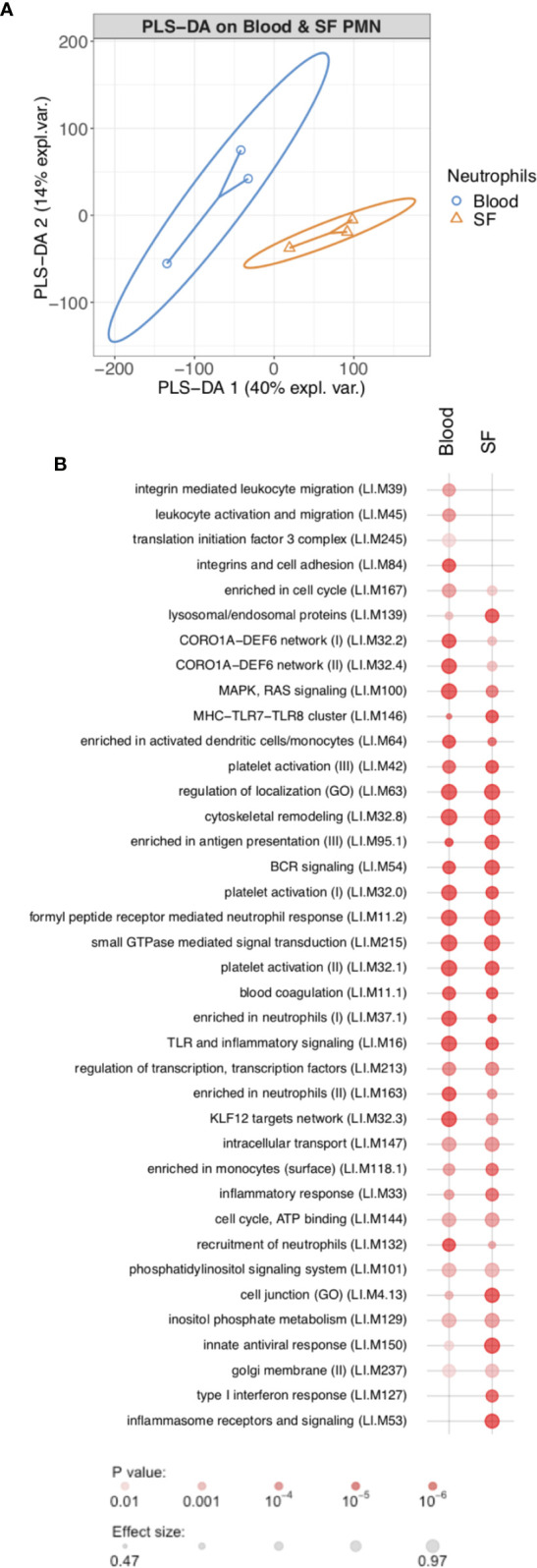
Transcriptomic analysis of rheumatoid arthritis peripheral blood and synovial fluid (SF) neutrophils (PMN). **(A)** PLS-DA modeling of blood and SF neutrophil transcriptomes using mixOmics. Blood and SF neutrophils were discriminated based on two components with an AUC of 1.0 (p<0.05). **(B)** Modular analysis of gene expression networks in blood and SF neutrophils using *tmod* (AUC>0.8).

In order to determine the changes in RA neutrophil transcriptome induced by migration from peripheral blood to inflamed, synovial joints, we isolated paired blood and synovial fluid neutrophils from patients with RA (n=3) with active disease (DAS28>5.1) prior to commencement of biologic therapy with the TNF inhibitor etanercept. Transcriptome sequencing (RNAseq) identified 772 genes that were significantly different between peripheral blood neutrophils and synovial fluid (SF) neutrophils (FDR<0.05). Of these, 412 genes were significantly higher in blood neutrophils and 347 were higher in synovial fluid neutrophils. Multivariate partial least squares-discriminant analysis (PLS-DA) on the expressed neutrophil transcriptome (~14,000 genes) modelled the difference between PB and SF neutrophil transcriptomes with a ROC AUC = 1 (**Figure 1**, p<0.05, two components). Modular analysis of the transcriptional networks active in RA blood and SF neutrophils revealed activation of gene modules regulating localization, cytoskeletal remodeling, and cell signaling (ATP, small GTPases, phosphatidylinositol) in both blood and SF neutrophils. However, signaling in response to MHC, toll-like receptors, inflammasomes and type I interferons was higher in SF neutrophils (**Figure 1B**). Modules corresponding to integrin signaling and recruitment of neutrophils were higher in blood neutrophils compared to SF.

**Figure 2:**
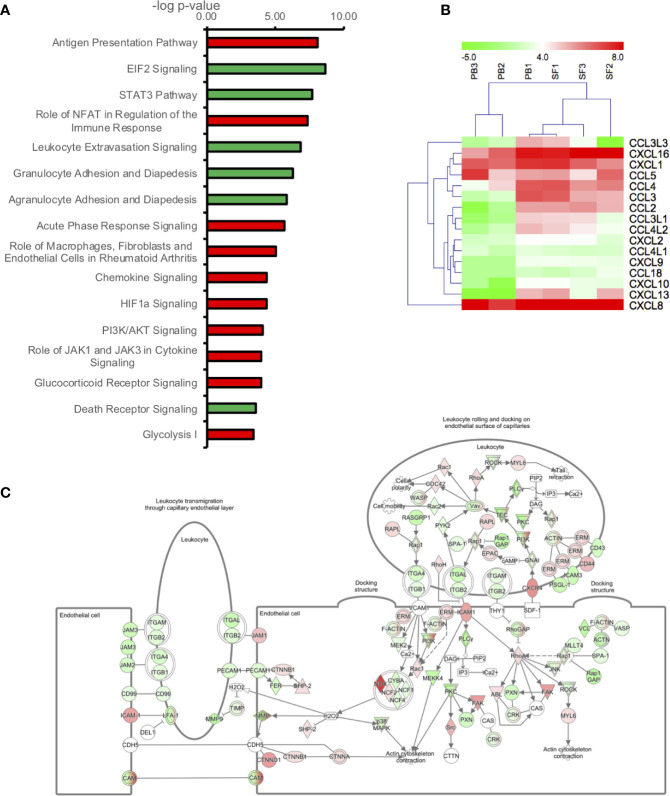
Transcriptomic analysis of genes differently expressed (1.5-fold change) between peripheral blood (PB) and synovial fluid (SF) neutrophils. **(A)** IPA signaling pathways predicted to be up-regulated (red) or down-regulated (green) in SF neutrophils. **(B)** Heatmap showing chemokine gene expression in PB and SF neutrophils. **(C)** Leukocyte extravasation pathway (p=1.41x10^-7^) showing genes up-regulated (red) or down-regulated (green) in SF neutrophils (white = not expressed).

In order to determine the signaling pathways most altered in RA neutrophils following migration from peripheral blood to synovial joints, we carried out bioinformatics analysis using Ingenuity Pathway Analysis (IPA) applying a 1.5-fold change in gene expression cut-off. The pathways most significantly up-regulated in SF neutrophils were: Antigen Presentation Pathway (p=10^-8^), Role of NFAT in the regulation of the immune response (p=10^-7^), and Acute phase response signaling (p=10^-6^). EIF2 Signaling (p=10^-8^), STAT3 pathway (p=10^-8^), Leukocyte Extravasation (p=10^-7^) and Granulocyte Adhesion and Diapedesis (p=10^-6^) were the most down-regulated pathways in SF neutrophils (**Figure 2A**). Chemokine signaling was up-regulated in RA SF neutrophils (p=10^-4^). Chemokines up-regulated in RA SF (**Figure 2B**) included those associated with attraction of both innate and adaptive immune cells, including other neutrophils (CXCL8, CXCL1, CXCL2), T cells, monocytes and macrophages, natural killer cells, and dendritic cells (CXCL16, CXCL10, CCL2, CCL3, CCL4) (31–33). Genes which were up- or down-regulated in SF and associated with the Leukocyte Extravasation Signaling pathway are shown in **Figure 2C** (p=10^-7^). HIF-1α signaling was up-regulated (p=10^-4^) in SF neutrophils, in line with the known hypoxic state of the RA SF joint (48), and the HIF1α transcription factor complex was predicted to be activated in RA SF neutrophils (p=5.8x10^-12^).

**Figure 3:**
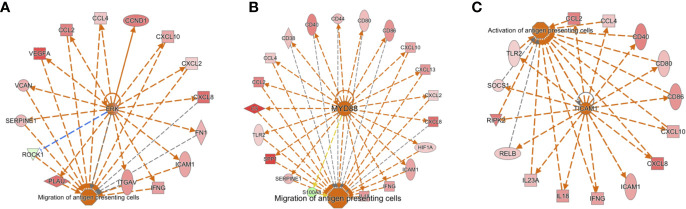
Activation of gene networks in RA SF neutrophils that control migration and activation of antigen presenting cells. These networks are regulated by **(A)** ERK, **(B)** MYD88, and **(C)** TICAM1.

The Antigen Presentation Pathway was predicted to be up-regulated (p=10^-8^), and indeed genes for both MHC Class I (B2M, HLA-A, HLA-B, HLA-C, HLA-G) and MHC Class II (HLA-DMA, HLA-DMB, HLA-DQB1, HLA-DRA, HLA-DRB1, HLA-DRB5) were all significantly higher in SF neutrophils, in line with previous reports (18). Networks of genes regulating the migration and activation of antigen presenting cells were predicted to be up-regulated in SF neutrophils (**Figure 3**). In particular, these networks were predicted to be regulated by ERK (**Figure 3A**), MYD88 (**Figure 3B**), and TICAM1 signaling (**Figure 3C**). We also noted genes encoding the high affinity FcγR1 (FCGR1A, FCGR1B, FCGR1C) were significantly higher in RA SF neutrophils (FDR<0.01), as previously reported (19).

Glycolysis enzymes were highly expressed in RA SF neutrophils (glycolysis pathway activation, p=10^-3^). This is in line with our recent metabolomics analysis of RA SF which identified lower levels of substrates for glycolysis in RA SF; this corresponds to higher levels of anaerobic cellular metabolism under hypoxic conditions (49). Lactate dehydrogenase enzymes were also higher in SF neutrophils (LDHA, LDHB; FDR<0.05). These enzymes catalyze the reduction of pyruvate to lactate producing NAD^+^ and enabling continued energy production under the hypoxic cellular state (50).

### Effect of Synovial Fluid on Neutrophil Migration, Apoptosis, and ROS Production

**Figure 4:**
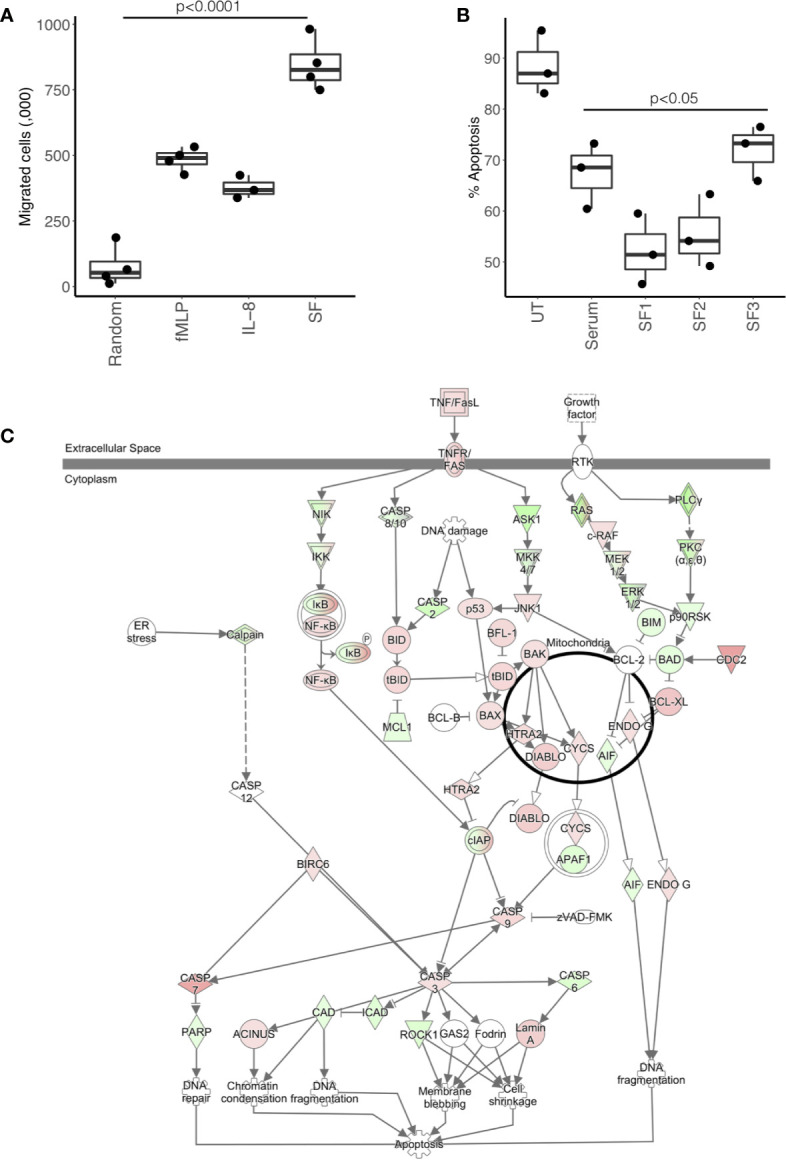
Effect of RA SF on neutrophil migration and apoptosis. **(A)** Neutrophil chemotaxis was significantly increased towards fMLP, interleukin-8 (IL-8) and 10% RA SF (p < 0.0001). **(B)** Neutrophil apoptosis was significantly delayed by RA SF over 18 h (p < 0.05). **(C)** IPA apoptosis signaling pathway showing up-regulation (red) and down-regulation (green) of genes associated with regulation of apoptosis in SF neutrophils (white = not expressed).

In order to validate the predictions of the IPA analysis, we tested the ability of RA SF to alter neutrophil function. We found that 25% v/v RA SF (three separate donors, all RF positive) could significantly induce healthy blood neutrophil migration through a 3 μM transwell membrane (**Figure 4A**, ANOVA p<0.0001) compared to both random migration (Tukey’s post-hoc p<0.001), and known chemoattractants fMLP (10^-8^M, Tukey’s post-hoc p<0.001) and interleukin-8 (IL-8, 100 ng/ml, Tukey’s post-hoc p<0.001). We also observed a delay in apoptosis (18 h) when we incubated healthy control neutrophils in 10% cell-free SF (**Figure 4B**). This delay in apoptosis was significant for all RA SF compared to untreated (media only) neutrophils (p<0.05). However, when the RA SF was compared to 10% AB serum, the delay in apoptosis was only significant for patient SF1 (p<0.05). Apoptosis was not significantly altered by migration into synovial fluid through a transwell membrane (3 μM, data not shown). IPA analysis indicated that this delay in apoptosis was *via* the up-regulation of the NFκB transcription factor complex (p=2.9x10^-8^) in response to activation of TNF receptors 1 and 2 (TNFR1 signaling p=10^-2^; TNFR2 signaling p=10^-3^) in RA SF neutrophils (**Figure 4C**).

**Figure 5:**
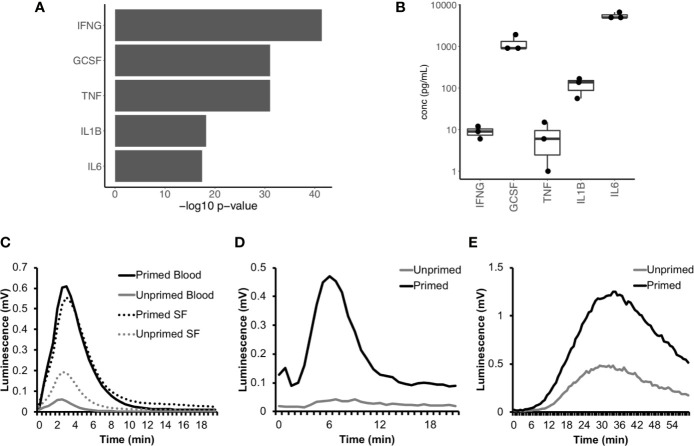
Cytokines in RA SF prime the neutrophil respiratory burst. **(A)** IPA predicted up-stream cytokine regulators of gene expression in RA SF neutrophils (IFNG, interferon-γ; GCSF, granulocyte colony stimulating factor; TNF, tumor necrosis factor α; IL1B, interleukin 1β; IL6, interleukin 6). **(B)** Levels of cytokines measured in RA SF from which the neutrophils sequenced by RNA-seq were isolated. **(C)** ROS production by RA blood and SF neutrophils (with and without cytokine priming) to fMLP. RA SF (25% v/v) also activates ROS production in healthy neutrophils. Stimulation of neutrophils with RA SF containing soluble immune complexes **(D)** activates ROS production in cytokine primed neutrophils only, whereas RA SF containing insoluble immune complexes **(E)** activates ROS production in both primed and unprimed neutrophils.

We next used IPA to predict which cytokines were regulating neutrophil gene expression in RA SF. The major cytokines predicted were interferon-gamma (IFNγ), TNFα, interleukin-1β (IL-1β), interleukin-6 (IL-6), and granulocyte-colony stimulating factor (G-CSF) (**Figure 5A**, p<10^-15^). The levels of these cytokines had previously been measured in the donor RA SF as part of a parallel study (**Figure 5B**) (47). Evidence of RA SF neutrophil activation by cytokines can be demonstrated by measuring the respiratory burst in cytokine-primed and unprimed RA blood and SF neutrophils using luminol-enhanced chemiluminescence. When unprimed blood neutrophils were activated with fMLP, little or no ROS production was observed; this was greatly enhanced by priming for 20 min with TNFα (**Figure 5C**). However, unprimed RA SF neutrophils produced around 2-fold greater ROS compared to unprimed RA blood neutrophils, indicating the SF neutrophils have been primed *in vivo* by synovial cytokines (**Figure 5C**). Production of reactive oxygen species by RA SF neutrophils was predicted by IPA canonical pathway analysis (p=10^-3^, **Supplementary Figure 2**).

IPA analysis also predicted that RA SF neutrophils had been activated by immunoglobulins (p<0.01, **Supplementary Figure 3**); this is likely to be immune complexes such as rheumatoid factor (RF) and/or anti-citrullinated protein antibodies (ACPA). The presence of immune complexes in RA SF can be demonstrated using luminol-enhanced chemiluminescence which detects ROS produced by cytokine-primed or unprimed neutrophils in response to the addition of RA SF (20). In our experiments, soluble immune complexes present in RA SF (25% v/v) activated a rapid respiratory burst in cytokine-primed neutrophils (but not unprimed neutrophils) (**Figure 5D**) whereas insoluble immune complexes present in RA SF (25% v/v) activated a slower and more sustained respiratory burst in both cytokine-primed and unprimed neutrophils (**Figure 5E**).

### Effect of Synovial Fluid on NET Production

**Figure 6:**
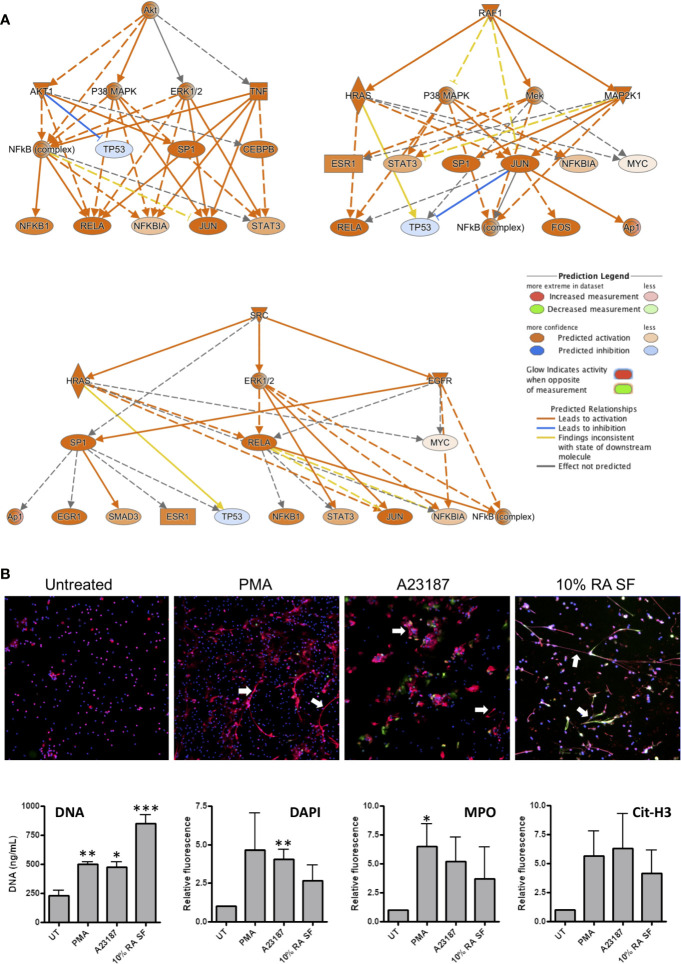
Activation of NET production by RA SF. **(A)** IPA predicted activation of signaling cascades regulated by AKT, RAF1 and SRC which may regulate NET production. **(B)** Neutrophils were incubated for 4 h with PMA (50 nM), A23187 (3.8 uM), or 10% RA SF. NET production was increased by RA SF as measured by quantification of extracellular DNA in culture supernatants, DAPI staining of extracellular NET DNA (blue), and immunofluorescent staining for myeloperoxidase (MPO, red) and citrullinated histone H3 (cit-H3, green) on NET structures (*p < 0.05, **p < 0.01, ***p < 0.001). White arrows indicate examples of NETs on each panel.

NETs are implicated in the pathology of RA by the exposure of antigenic proteins such as citrullinated histones (1, 25). Whilst the exact signaling mechanisms regulating NET production in have yet to be fully elucidated, several signaling pathways that contribute to NET production have been proposed, including: Raf-MEK-ERK, RIPK1-RIPK3-MLKL, AKT, p38-MAPK and cSrc (51–54). Interestingly, all these signaling pathways were predicted by IPA as up-stream regulators of gene expression in RA SF neutrophils (p<0.01). Signaling networks regulated by AKT, RAF1, and SRC are shown in **Figure 6A**.

We propose that activation of these kinases in RA SF neutrophils will lead to NET production, and therefore tested the ability of RA SF to activate NET production by healthy control neutrophils. We found that 10% RA SF increased NET production by neutrophils to levels similar to that of positive controls PMA and A23187 as shown by the concentration of DNA in culture supernatants (p<0.001), as well as the level of extracellular DNA (DAPI), myeloperoxidase (MPO) and citrullinated histone H3 visible on exposed on immunofluorescently labeled NET structures (**Figure 6B**).

## Discussion

In this study we have described for the first time the changes in gene expression that take place in RA neutrophils following migration from peripheral blood into inflamed joints. Using RNA-seq we have revealed an activated neutrophil phenotype in RA SF, characterized by increased expression of chemokines, delayed apoptosis and increased activation of kinases and transcription factors which may be implicated in the production of NETs.

Our experiments showed that RA SF decreased the rate of neutrophil apoptosis, which may be attributed to the high levels of inflammatory cytokines present in SF. We previously measured the levels of 13 cytokines in RA SF and found high levels of IL-1β, IL-1ra, IL-2, IL-4, IL-8, IL-10, IL-17, IFN-γ, G-CSF, GM-CSF, and TNF-α (47). Many of these cytokines have been demonstrated individually to delay neutrophil apoptosis *in vivo*, including G-CSF, GM-CSF, IL-1β, TNFα, and IFN-γ (55–62), although this is often at concentrations in excess of those found *in vivo*. GM-CSF secreted by synovial fibroblasts in response to IL-17 and TNFα has been shown to delay neutrophil apoptosis (63), however a separate study found that apoptosis delay induced by RA SF was not related to the TNF or GM-CSF content, but did correlate with adenosine (64). Lactoferrin present in RA SF also delays neutrophil apoptosis and may serve as a feed-back anti-apoptotic mechanism for activated neutrophils (10). In a separate study, RA SF was shown to be pro-apoptotic in overnight neutrophil cultures, but this effect was reversed when neutrophils were incubated with SF under conditions of hypoxia which more closely model the RA joint (48). Relatively few apoptotic neutrophils are present in freshly-isolated RA SF (65), and while SF is anti-apoptotic, the question remains as to the fate of SF neutrophils. One possibility is that they undergo efferocytosis by synovial macrophages, or another is that they may reverse migrate from the joint back into circulation, as demonstrated in zebrafish models of inflammation (66). The ability of *ex vivo* RA SF neutrophils to migrate in response to chemoattractants such as IL-8 *in vitro* should be investigated in future experiments.

RA neutrophils contain high intracellular levels of citrullinated proteins, including known auto-antibody targets: cit-actin, cit-histone H1.3, cit-histone H3, cit-vimentin (25, 67). Several of these citrullinated auto-antigens are present on RA NETs (45). The RAGE-TLR9 pathway plays a key role in both the internalization and presentation of citrullinated NET peptides on MHC Class II in fibroblast-like synoviocytes (FLS). This leads to the development of ACPA specific to the citrullinated NET peptides and cartilage damage in mouse models of RA (68). A role for NET-derived elastase in cartilage destruction has also been proposed, whereby elastase contained in NET material disrupts the cartilage matrix and induces the release of PAD2 by fibroblast-like synoviocytes (FLS) leading to citrullination of cartilage fragments (69). These citrullinated cartilage fragments are subsequently internalized and presented by FLS and macrophage to antigen specific T-cells leading to the development of auto-immunity and ACPA in HLA-DRB1*04:01 transgenic mice (69).

Recent proteomic analysis of SF from RA patients and spondyloarthritis (SpA) patients identified elevated levels of many neutrophil proteins in RA SF, including MPO, cathepsin G, annexin-A1 and NGAL. Interestingly, whilst the concentration of cell-free DNA did not differ between RA and SpA SF, the levels of 21 NET proteins were elevated in RA SF, including histones H2A, H2B and H4, MMP9, elastase, and α-enolase (12). It has previously been shown that levels of ACPA in RA SF correlate with neutrophil numbers and severe disease activity, and that SFs with high ACPA titers induce high levels of ROS and NET production (70). A separate study demonstrated that depletion of ACPA from RA SF inhibits NET production *in vitro* (71). A role for IgA immune complexes in RA SF has also been proposed, with SF rich in IgA inducing NET and ROS production, and release of lactoferrin by healthy control neutrophils in a mechanism that was inhibited by blockade of the FcαRI receptor (72). RA SF also contains high levels of extracellular DNA, concentrations of which correspond to neutrophil counts and PAD activity, and which have been attributed to NETs (27). Our recent proteomics study identified both PAD2 and PAD4 in RA NETs (45). PAD enzymes have also been identified by IHC in synovial biopsies, localized with MPO in necrotic areas of synovial tissue (6) that contain large areas of citrullinated and hypercitrullinated proteins, possibly indicating the presence of NET structures within synovial tissue.

Whilst the exact signaling mechanism(s) regulating NET production remain unclear, at least two types of NET production (NETosis) have been described: NOX2-dependent and NOX2-independent. NOX2-dependent NETosis occurs *via* activation of the NADPH-oxidase (NOX2) and production of ROS. This causes increased intracellular membrane permeability, elastase release into the nucleus and degradation of histones leading to chromatin decondensation and NET release (73). NOX2-independent NETosis does not require the production of ROS by the NADPH oxidase. In this case, mitochondrial ROS combine with increased intracellular calcium levels to activate PADs leading to hypercitrullination of histones, chromatin decondensation and NET release (74, 75). Signaling pathways including Raf-MEK-ERK, RIPK1-RIPK3-MLKL, AKT, p38-MAPK, and cSrc have been identified as some of the drivers of NETosis (51–54). In our study, all of these signaling pathways were predicted to be activated in RA SF neutrophils. Interestingly, all of the kinase activation networks predicted also included downstream activation of NFκB. This important signaling pathway, activated by cytokines such as TNFα and IL-1β, has not yet been implicated in NET production and would be an interesting candidate for future investigation, which should focus on the use of signaling inhibitors to understand the regulation of NET production in RA blood and SF neutrophils. Recent work has shown that the production of NETs by inflammatory neutrophils leads to a forward-feedback loop, where NET debris (DNA:protein complexes) further activates surrounding neutrophils *via* toll-like receptors (TLRs) 7, 8, and 9 (76). RNA:protein complexes have also been identified within human neutrophil NETs. Furthermore, RNA:LL37 complexes from NETs induced NET release and expression of cytokines (TNFα, IL-6, IL-1β, CCL4) *via* TLR8 (77). Interestingly, our informatics prediction of gene networks activating SF neutrophils identified MyD88 and TICAM1 (both downstream components of TLR signaling (78)) as key signaling proteins activated in RA SF neutrophils. This suggests that SF neutrophils may be activated by NET debris within RA SF, something that should be the focus of future investigation.

**Figure 7:**
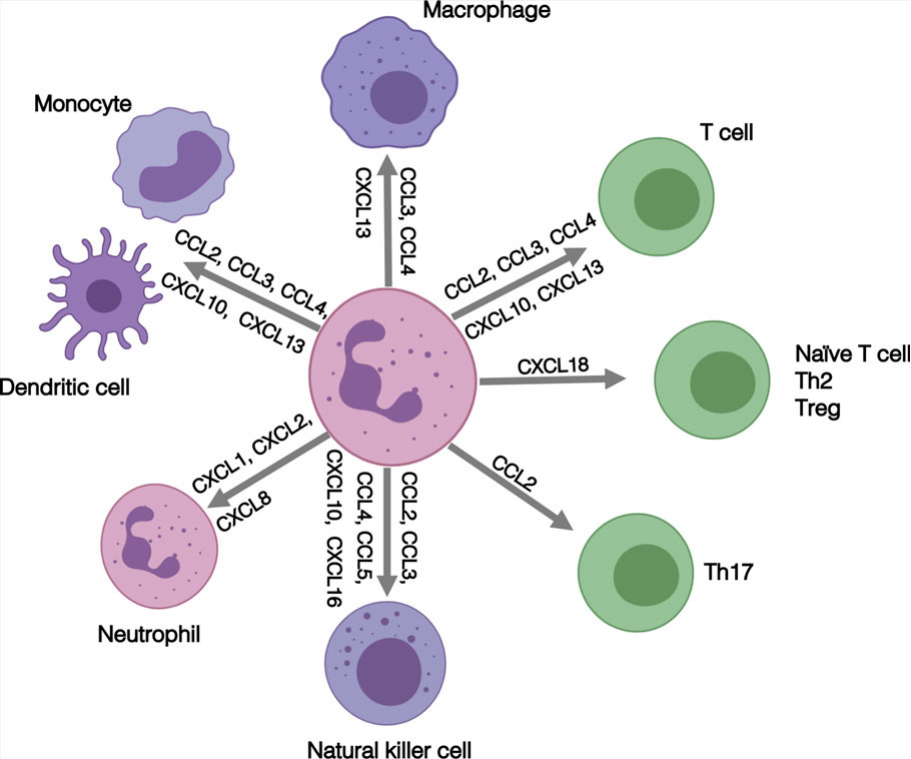
Summary of the role of inflammatory role of chemokines expressed by RA SF neutrophils.

Our RNA-seq analysis identified increased expression of MHC Class II genes in RA SF neutrophils. We and others have previously shown that whilst healthy control neutrophils do not express MHC Class II, activated neutrophils can express both MHC Class II mRNA and protein and stimulate proliferation of T cells (18, 79–81) RA blood neutrophils express MHC Class II RNA, and RA SF neutrophils expression both RNA and MHC Class II protein, although the latter was contained within intra-cellular pools which were mobilized to the plasma membrane following overnight incubation (18). While expression of co-stimulator molecules CD80 and CD86 were detected only at very low levels, SF neutrophils were able to stimulate CD4^+^ T-cell proliferation *via* a mechanism that was inhibited by an anti-MHC Class II antibody (18). Our transcriptomics analysis also detected increased expression of a number of chemokines in RA SF (shown in **Figure 2B**), including CCL3, CCL4, CCL10, CXCL16, CXCL2, and CXCL8. These chemokines play a key role in regulating the inflammatory response in the joint, not only through recruitment of other neutrophils (CXCL1, CXCL2, CXCL8), but also through the recruitment and activation of both innate and adaptive immune cells (31–33) as summarized in **Figure 7**. This increased production of chemokines within the joint, coupled with a down-regulation of adhesion receptors, suggests that RA SF neutrophils become resident within the joint to drive further inflammation through recruitment and activation of other immune cells.

Our findings provide novel insight into the multitude of ways that synovial neutrophils drive chronic inflammation in RA. This raises the possibility that aberrant neutrophil activation may be a target of future therapeutics in this chronic and life-limiting condition. A number of new therapies which directly or indirectly target neutrophil function have been proposed or are in clinical trial (82). Inhibitors of CXCR1/CXCR2, the receptor for CXCL8 (interleukin-8), have been demonstrated to reduce neutrophil adhesion and recruitment to synovial joints in murine models of inflammatory arthritis (83), an effect which was associated with lower TNFα levels and disease activity (84). JAK inhibitors including tofacitinib and baricitinib, which target signaling down-stream of cytokine receptors including IL-6, interferon-α and -γ, and GM-CSF receptors, are clinically effective in treating RA (85, 86) although many patients report transient neutropenia and increased infections. These drugs have been shown to inhibit cytokine signaling in neutrophils, including inhibition of migration and ROS production by RA neutrophils (87). The anti-GM-CSF therapy mavrilimumab was effective in decreasing RA disease activity in a Phase 2b clinical trial, with over 70% of RA patients achieving an ACR20 improvement in the group receiving the highest dosing regime (88). Anti-G-CSF therapy prevents neutrophil migration into joints, suppressing cytokine production and halting the progression of murine arthritis (89). Finally, a number of novel therapeutics which target neutrophil proteases, including MPO and elastase, have been effective in reducing neutrophil-driven inflammation in animal models of inflammatory disease (90) and human respiratory disease (91) respectively. These inhibitor drugs have potential to inhibit NET production and damage to cartilage associated with inflammation in RA synovial joints, and thus may be potential therapeutics for repurposing to target neutrophil-driven RA joint inflammation.

One limitation of our study is the use of an *in vitro* model of RA to validate our transcriptomics data. Whilst the incubation of healthy control neutrophils in RA SF is a widely used model of the disease (48, 70–72), future studies should focus on validating the RA SF neutrophil phenotype using *ex vivo* RA SF neutrophils. There have been a number of reports on the properties of RA SF neutrophils and in general these show an activated phenotype, in terms of surface receptor expression, ROS production and apoptosis (see *Introduction*). We show here for the first time that RA SF neutrophils also have an altered transcriptome compared to blood neutrophils, and that analysis of these transcriptional changes reveals that RA SF neutrophils not only have altered functions but are activated to express a range of chemokines that can exacerbate inflammation by the recruitment of more neutrophils and other leukocytes. While we have only analyzed the transcriptomes of paired blood and SF neutrophils from three patients with active disease, the data between these samples are consistent and entirely in line with other functional changes that have been measured. However, it would be interesting to expand this study to measure transcriptome changes in RA patients with different severity of disease and post-therapy to determine if disease improvement and resolution of inflammation is associated with reversal of these transcriptional signals.

In addition, future experiments should clarify whether the RA SF phenotype reported in our study is specific to inflammation within the RA joint, or whether this neutrophil phenotype is more reflective of a more generalized inflammatory activation. Neutrophils have been shown to be transcriptionally plastic during migration from bone marrow to peripheral blood and into inflammatory tissue. In a mouse model of peritoneal inflammation around a thousand neutrophil genes were differentially regulated during migration between these three compartments (92). Interestingly a number of chemokine and cytokine genes, including CCL3, CXCL1, CXCL2, and CXCL3, were increased in peritoneal neutrophils following migration. This is in line with our observation of an increase in chemokine production following migration to inflamed joints. These transcriptional changes during migration into the inflamed peritoneum were also mirrored by an increase in expression of anti-apoptotic genes (92). This suggests that at least some of the transcriptional changes we observed in our study are associated with the physical movement of neutrophils from peripheral blood into inflamed tissues, possible due to integrin signaling during adhesion and the physical act of squeezing and migrating through the endothelial layer of blood vessels and the basement membrane of connective tissues. This requires further investigation, although is challenging to test in a human model. Further work to examine the phenotype of synovial joint neutrophils from conditions such as gout or reactive arthritis would go some way to answering this question. Finally, further investigation of the signaling pathways regulating apoptosis and the production of NETs by RA SF, and the effect of small molecule inhibitors of intracellular signaling, would potentially identify novel therapeutic targets of NET production that would specifically target neutrophil activation within the RA joint.

In conclusion, we have used RNA-seq and experimental analysis of paired blood and SF RA neutrophils to describe a pro-inflammatory SF neutrophil phenotype which includes delayed apoptosis, production of ROS, and release of NETs. RA SF neutrophils down-regulate adhesion molecules to become resident in the joint and drive inflammation *via* increased production of chemokines that attract and activate both innate and adaptive immune cells. We propose this altered neutrophil phenotype contributes to the pro-inflammatory nature of active RA and explains the role of neutrophils in the pathogenesis of the disease.

## Data Availability Statement

The datasets presented in this study can be found in online repositories. The names of the repository/repositories and accession number(s) can be found in the article/**Supplementary Material**.

## Ethics Statement

The studies involving human participants were reviewed and approved by University of Liverpool Committee on Research Ethics for healthy controls, and NRES Committee North West (Greater Manchester West, UK) for RA patients. The patients/participants provided their written informed consent to participate in this study.

## Author Contributions

HW designed the research, carried out the experiments, analyzed the data, and wrote the manuscript. ML carried out the experiments and analyzed the data. EC carried out the experiments, analyzed the data and revised the manuscript. RM revised the manuscript. SE analyzed the data and revised the manuscript. All authors contributed to the article and approved the submitted version.

## Funding

HW was funded by a Versus Arthritis Career Development Fellowship (No. 21430). EC was funded by a Wellcome Trust Seed Award in Science (No. 200605/Z/16/Z). ML was funded by the University of Liverpool MRes Clinical Sciences Research Support Fund. The Epifluorescence microscope at Liverpool Centre for Cell Imaging was funded by MRC grant number MR/K015931/1.

## Conflict of Interest

The authors declare that the research was conducted in the absence of any commercial or financial relationships that could be construed as a potential conflict of interest.
