# Supplementary material for: Rheumatoid Arthritis Synovial Fluid Neutrophils Drive Inflammation Through Production of Chemokines, Reactive Oxygen Species, and Neutrophil Extracellular Traps
Source: Front Immunol. 2021 Jan 5;11:584116. doi: 10.3389/fimmu.2020.584116 (PMC7813679; doi:10.3389/fimmu.2020.584116)
Supplement: Supplementary file 1 [file DataSheet_1.docx]

**Rheumatoid arthritis synovial fluid neutrophils drive inflammation through production of chemokines, reactive oxygen species and neutrophil extracellular traps**

Helen L Wright^1^, Max Lyon^1^, Elinor A Chapman^1^, Robert J Moots^2,3^, Steven W Edwards^4^

^1^Institute of Life Course and Medical Sciences, University of Liverpool, Liverpool, UK.

^2^Department of Rheumatology, Aintree University Hospital, Liverpool, UK.

^3^Faculty of Health, Social Care and Medicine, Edge Hill University, Ormskirk, UK.

^4^Institute of Infection, Veterinary and Ecological Sciences, University of Liverpool, Liverpool, UK.

Supplementary Material

# Supplementary Data

**Supplementary Figure 1.** Cytospin images of purified blood neutrophils (PMNs), whole SF, and purified SF neutrophils.


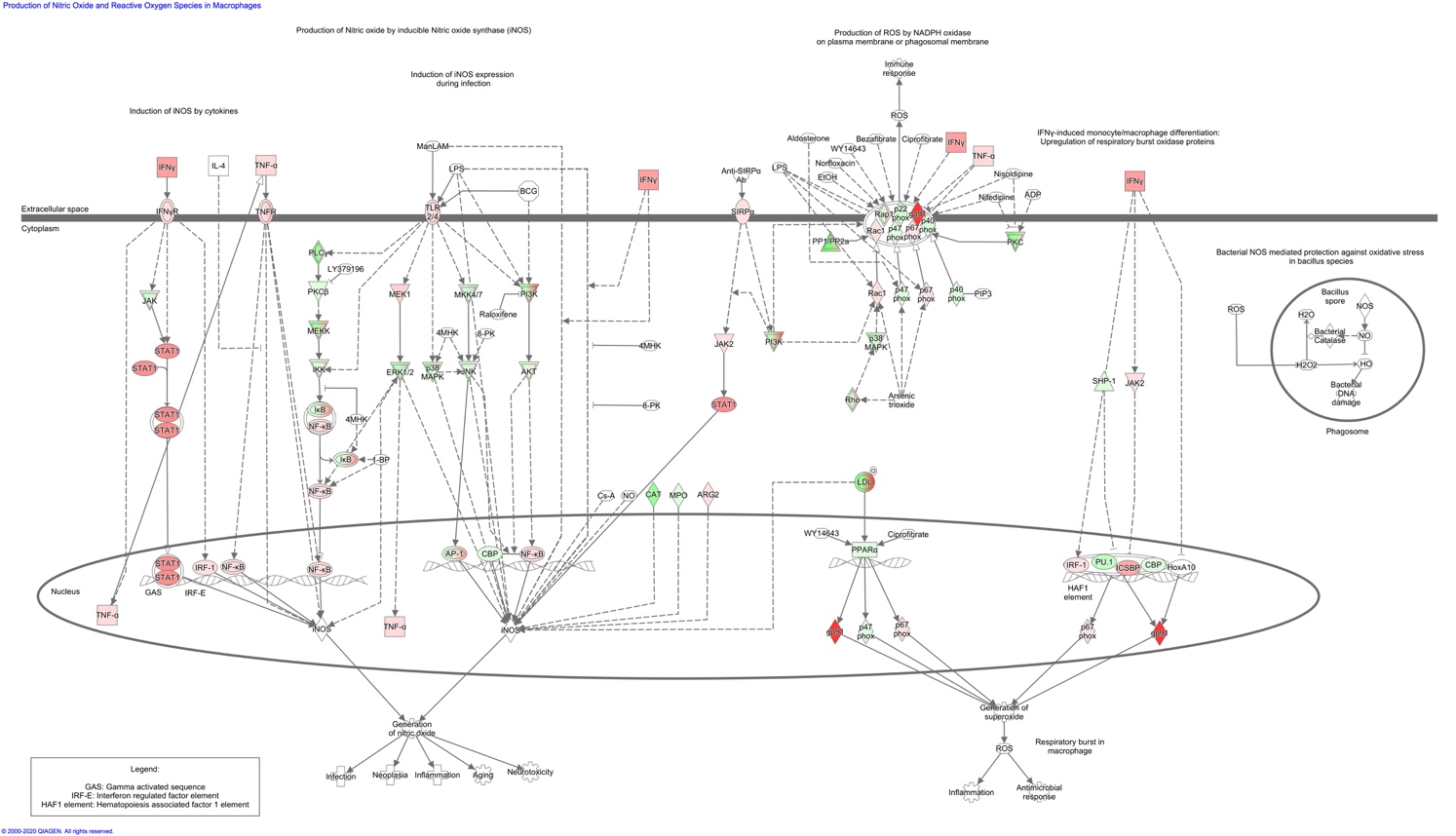


**Supplementary Figure 2.** Canonical pathway relating to production of nitric oxide and reactive oxygen species in macrophages was predicted by IPA to be activated in RA SF neutrophils (p=10^-3^).


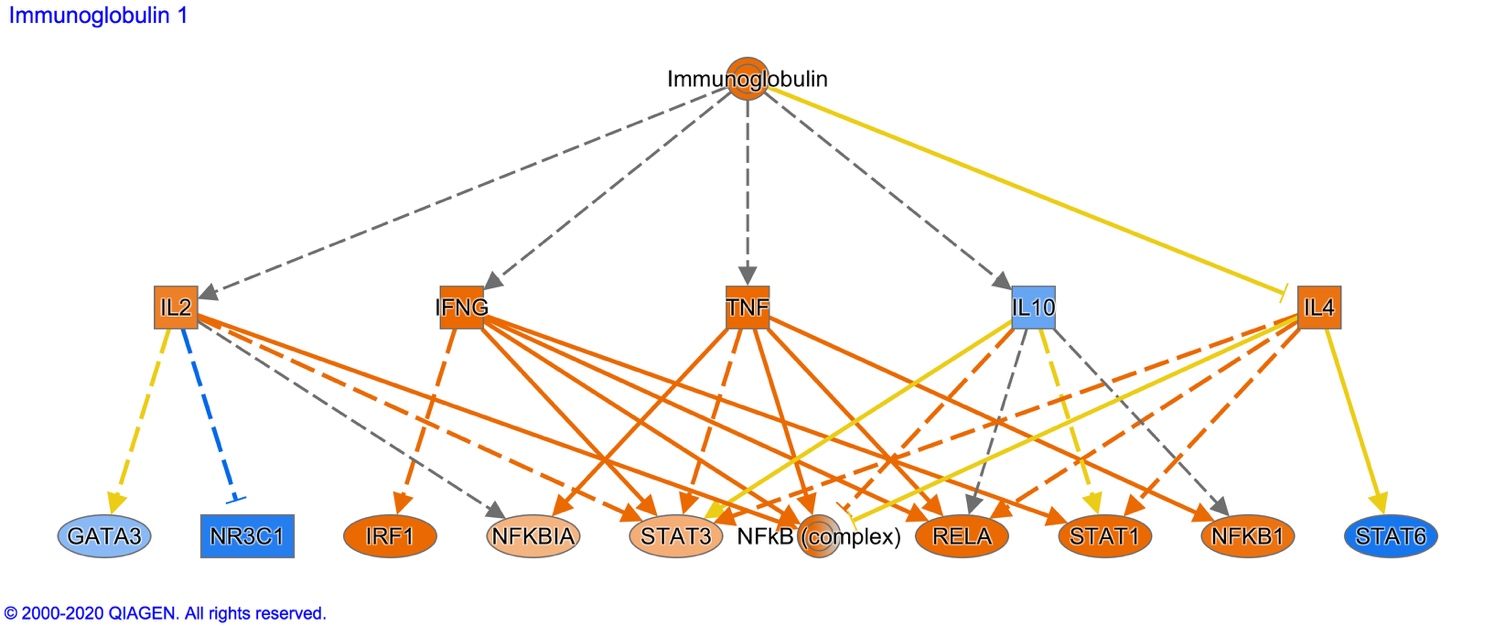


**Supplementary Figure 3.** Gene network predicted by IPA to be activated by immunoglobulins in RA SF neutrophils (p<0.01).

**Supplementary Table 1.** Clinical characteristics of patients included in the study. (* % neutrophils in SF assessed by cytospin morphology).

| Patient ID | Age (Y) | Gender | Disease duration (Y) | CRP (g/L) | ESR (mm/h) | RF status | DAS28 score | % neutrophils in SF* |
| --- | --- | --- | --- | --- | --- | --- | --- | --- |
| 1 | 59 | M | 20 | 49 | 22 | + | 5.10 | 76.4 |
| 2 | 74 | F | 17 | 212 | 69 | + | 7.56 | 86.6 |
| 3 | 44 | F | 25 | 64 | 36 | - | 5.52 | 93.8 |
